# Immunomodulatory Effects of *Escherichia coli* ATCC 25922 on Allergic Airway Inflammation in a Mouse Model

**DOI:** 10.1371/journal.pone.0059174

**Published:** 2013-03-25

**Authors:** Wenhui Pang, Hefeng Wang, Lei Shi, Yueqi Sun, Xiaoting Wang, Mingming Wang, Jianfeng Li, Haibo Wang, Guanggang Shi

**Affiliations:** 1 Institute of Eye and Otorhinolaryngology, Department of Otorhinolaryngology-Head and Neck Surgery, Provincial Hospital affiliated to Shandong University, Jinan, China; 2 Qilu Hospital, Shandong University, Jinan, China; 3 Otorhinolaryngology Hospital, The first affiliated hospital, Sun Yat-Sen University, Guangzhou, China; French National Centre for Scientific Research, France

## Abstract

**Background:**

Hygiene hypothesis demonstrates that the lack of microbial exposure would promote the development of allergic airway disease (AAD). Therefore, the gut microbiota, including *Escherichia coli* (*E. coli*), would probably offer a potential strategy for AAD.

**Objective:**

To investigate whether *E. coli* infection is able to suppress the induction of AAD and to elucidate the underlying mechanisms.

**Methods:**

Nonpathogenic *E. coli* ATCC 25922 was infected by gavage before AAD phase in three patterns: 10^8^ or 10^6 ^CFU in neonates or 10^8 ^CFU in adults. Then mice were sensitized and challenged with ovalbumin (OVA) to induce allergic inflammation in both the upper and lower airways. Hallmarks of AAD, in terms of eosinophil infiltration and goblet cell metaplasia in subepithelial mucosa, Th2 skewing of the immune response, and levels of T regulate cells (Tregs), were examined by histological analysis, ELISA, and flow cytometry, respectively.

**Results:**

*E. coli*, especially neonatally infected with an optimal dose, attenuated allergic responses, including a decrease in nasal rubbing and sneezing, a reduction in eosinophil inflammation and goblet cell metaplasia in subepithelial mucosa, decreased serum levels of OVA-specific IgE, and reduced Th2 (IL-4) cytokines. In contrast, this effect came with an increase of Th1 (IFN-r and IL-2) cytokines, and an enhancement of IL-10-secreting Tregs in paratracheal lymph nodes (PTLN).

**Conclusion:**

*E. coli* suppresses allergic responses in mice, probably via a shift from Th1 to Th2 and/or induction of Tregs. Moreover, this infection is age- and dose-dependent, which may open up novel possibilities for new therapeutic interventions.

## Introduction

Allergic airway disease (AAD), such as allergic rhinitis, asthma, and so forth, is reversible and chronic atopic disorders, resulting from complex immunological interactions between genetic susceptibility and environmental factors [1], [2]. More recently, mounting body evidence illustrates that the upper and lower airways share common pathologies and mechanisms, accounting for allergen specific T-helper (Th) 2 lymphocyte proliferation with concomitant excessive Th2 cytokines interleukin (IL)-4 and so forth [3], [4]. The skewing of Th2-like immune responses also includes eosinophil inflammation and goblet cell metaplasia in subepithelial mucosa, as well as increased serum levels of allergen-specific immunoglobulin (Ig) E, all synthetically contributing to allergic airway inflammation [5]–[7]. In recent decades, the prevalence of AAD has dramatically increased worldwide, drawing global public health attention [1], [2]. Meanwhile, there are notable disparities in the prevalence of allergic rhinitis and asthma between developed and developing countries, and between urban and rural areas in the same country [8]–[9].

Hygiene hypothesis has demonstrated that the overly hygienic lifestyle leads to a gradual disappearance of the gut microbiota, thus disturbing the balance of our immune system and contributing to AAD epidemic [8]–[11]. Although the exact mechanism has not been well understood, growing researches in those areas including epidemiology, experimental and preliminary clinical studies [12]–[14] have all demonstrated that exposure to certain microbiota or their products during neonatal or early childhood possess important and far-reaching significance for the prevention and protection against AAD. Recently, Blaser and Falkow [15] declared that it was the lack of our ancestral indigenous microbiota associated with the prevalence of AAD, rather than a general decline in arbitrary infections. Additionally, some studies [16], [17] postulated that the mucosal surfaces of the nose, the lung and the gut were particularly interacted because they were predominant sites of microbial infection, and jointly played a role in modulating allergic responses. For this reason, the gut microbiota has been inferred to possess strong immunomodulatory properties negatively associated with allergic airway inflammation.

*Escherichia coli* (*E. coli*) is the main and most prevalent gut microbiota that persistently colonizes in the intestine of human and many animals known as enterobacteriaceae [18], whose rapid disappearance is epidemiologically linked to the development of allergic diseases [19], [20]. Actually, *E. coli* consists of a diverse group of bacteria, most of which is harmless and an important part of a healthy human intestinal tract by producing vitamin K2 and by preventing the establishment of pathogenic bacteria [21]. *E. coli* ATCC 25922 is a nonpathogenic strain of *E. coli,* which is BSL-1 certified to make it useful for various laboratory experiments [22]. It is not only well-characterized as a control Gram-negative bacterium, but most widely studied as a prokaryotic model organism in the fields of biotechnology and microbiology served as the host organism [23], [24].

As yet, to the best of our knowledge, no study has been conducted to elucidate the contribution of *E. coli* in vivo to allergic rhinitis and/or asthma. Therefore, herein we adopted *E. coli* ATCC25922 by three approaches of intestinal infection prior to ovalbumin (OVA)-induced allergic airway inflammation in mice, examined its immunomodulatory efficacy, as well as elucidated the underlying mechanisms. We aimed to provide experimental evidence for the beneficial effect of *E. coli* against AAD, and to consider how our knowledge of these inverse interactions to be harnessed to improve people’s health.

## Materials and Methods

### Animals and Reagents

Specific pathogen free (SPF) female Balb/c mice when 4∼5 days and 4∼5 weeks of age were obtained from Shandong University School of Medicine (Jinan, China) and maintained under SPF conditions with free access to sterile water and food in individual ventilated cages. All experiments were approved by the Animal Care Committee of Shandong University (NO. ECAESDUSM 20123011).

*E. coli* (ATCC, 25922, USA) was used in this study for in vivo experimentation. OVA (Sigma-Aldrich, A5503, USA) as the allergen, and aluminum hydroxide (Thermo Scientific Imject Alum, 77161, USA) as the immunologic adjuvant were obtained for sensitization & challenge. Staining reagents of Wright-Giemsa and alcian blue-periodic acid Stiff come from YiLi Bioscience and Technology (Beijing, P.R. China). Serum OVA-specific IgE (Chondrex Assay Kit, 3010, USA) was assayed to aid the diagnosis of the allergic state. Mouse IL-4, IL-10, IFN-gamma (IFN-γ) and IL-2 enzyme-linked immunosorbent assay (ELISA) kits (eBioscience, 88-7711, USA) and regulatory T cell staining kit (eBioscience, 88-8115, USA) were all purchased from eBioscience.

### Study Design for Infection, Sensitization and Challenge

**Figure 1:**
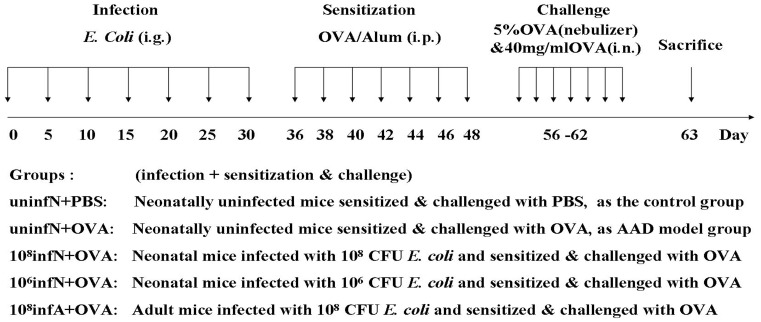
The experimental protocol and different groups. Mice were infected with *E. coli* or PBS, and then sensitized & challenged with OVA or PBS, as described previously in Materials and methods. Mice were divided into five different groups in this work.

To assay the effects of *E. coli* on OVA-induced allergic inflammation, Balb/c mice were divided into five groups (Fig. 1): (A) (uninfN+PBS) as the control group, neonatally uninfected mice were sensitized & challenged with PBS instead. (B) (uninfN+OVA) as AAD model group, neonatally uninfected mice were sensitized & challenged with OVA. (C) (10^8^infN+OVA) group, neonatal mice were infected with 10^8^ colony-forming units (CFU) *E. coli* by gavage and then sensitized & challenged with OVA. (D) (10^6^infN+OVA) group, neonatal mice were infected with a lower dose of oral 10^6^ CFU *E. coli* and then sensitized & challenged with OVA. (E) (10^8^infA+OVA) group, adult mice when 4∼5 weeks of age were infected with oral 10^8^ CFU *E. coli* and then sensitized & challenged with OVA. In our preliminary experiments, we have performed the control group of the adult mice, but we did not find any statistical differences between the controls of the neonatal mice and that of the adult mice in pathologic analysis or immune responses. Considering that there were no positive results between the controls, thus we dropped the assessment of the controls of adult mice in our study. Each experimental group consisted of 10 animals.

The detail experimental protocol was as following: without on need of anesthesia, mice in a sober state of nature were intragastric (i.g.) administrated with 100 µl volume of live *E. coli* ATCC 25922 on days 0, 5, 10, 15, 20, 25 and 30, using an 8-gavage needle tightly connected to a 1 ml syringe interface. Mice were infected with approximately 10^8^ or 10^6 ^CFU live *E. coli* as neonates (10^8^infN, 10^6^infN) or 10^8 ^CFU as adults (10^8^infA), along with two neonatally uninfected (uninfN) groups with PBS by gavage instead. After 5 days following *E. coli* treatment, mice were induced allergic airway inflammation as previously described with slight modification [25], [26]. All mice apart from one neonatally uninfected group were sensitized with 200 µl volume of 40 µg OVA & 2 mg Alum dissolved in PBS by intraperitoneal (i.p.) injection on days 36, 38, 40, 42, 44, 46 and 48, and then observed for 1 week. Hereafter on days from 56 to 62, mice in a plexiglas exposure chamber were challenged by inhalation of 5% OVA aerosols generated by an ultrasonic nebulizer at a flow rate of 6 L/min for 30 min, and then immediately were intranasally (i.n.) infused with 20 µl 5% OVA each day. Mice in the separate neonatally uninfected group were sensitized & challenged by PBS instead of OVA. After 24 h of the final challenge, all mice were sacrificed to collect samples.

### Measurement of Allergic Symptoms

Allergic symptoms were evaluated by counting the frequency of nasal rubbing and sneezing per mouse for 10 min, immediately after the last OVA challenge in a blinded manner by five observers as previously described [27].

### Cell Counts for Nasal Lavage Fluid (NALF) and Bronchoalveolar Lavage Fluid (BALF)

Mice were sacrificed after 24 h of the final allergen challenge. The nasal sections from nasopharynx to the nostril, and the lungs, were perfused with 0.4 ml×3 times PBS containing 1% fetal bovine serum (FBS) after partial tracheal resection using 22-gauge catheters, and then NALF and BALF were collected. Lavage fluids were centrifuged at 2500 rpm for 7 min at 4°C. After centrifugation, lavage supernatant of each mouse was separated and stored at −80°C until the further analysis. Lavage cells were collected and resuspended in 150 µl PBS containing 1% FBS. Total lavage cell numbers were evaluated using a hemocytometer. For different cell counts, cytospin preparations were made and stained with Wright-Giemsa, and differentiated into monocytes, eosinophils, lymphocytes, and neutrophils by standard morphology [28]. At least 300 cells of per cytospin preparation were counted at ×400 magnification under a light microscope (Leica, USA) and absolute numbers of every cell type were calculated.

### Hematoxylin and Eosin (HE) Staining for the Nasal Mucosa and Lung

After 24 h of the final challenge, all animals were sacrificed using a lethal dose of 10% chloral hydrate. The nasal tissues and lung were removed per mouse and fixed in 10% neutral buffered formalin for 36 h. After fixation, the nasal tissues still needed to be decalcified with 10% EDTA solution for 1 week. Samples were embedded in paraffin, and sections were all prepared at a thickness of 4 µm.

Sections of the nasal tissues, which were coronally at a distance of 5 mm from the nasal vestibule, were stained with HE to calculate numbers of eosinophils in subepithelial mucosa of the nasal septum through a light microscope at ×400 magnification. Inflammation scores in the lung were conducted using a reproducible scoring system as previously described [29]. Briefly, scores were set up and ranged from 0 to 3 based on the levels of peribronchial and perivascular inflammation across main bronchus. The values were given as follows: 0 for no inflammation; 1 for occasional cuffing with inflammatory cells; 2 for most bronchi or vessels surrounded by thin layer (1 to 5 cells) of inflammatory cells, and 3 for most bronchi or vessels were surrounded by a thick layer (more than five cells) of inflammatory cells. For quantifying numbers of eosinophils in the nasal mucosa and lung, five tissue sections of per mouse were randomly selected and carefully assessed in a blinded fashion.

### Alcian Blue and Periodic Acid Schiff (AB-PAS) Staining for the Nasal Mucosa and Lung

Sections of the nasal tissues and the lung were stained with AB-PAS to evaluate goblet cell metaplasia in the airway mucosa. Goblet cells were counted as the blue cells stained positive by AB-PAS and percentages were calculated from absolute numbers of cells counted around each airway by using a microscope as previously described [30]. For quantifying goblet cell metaplasia, percentages of AB-PAS positive cells in epithelial areas were assayed from five randomly selected tissue sections of per mouse in a blinded fashion.

### ELISA for Serum OVA-specific IgE

At 24 h after the last OVA challenge, blood was withdrawn from all mice via cardiac puncture to prepare serum for the measurement of OVA-specific IgE levels by ELISA as the manufacturer’s guideline.

### ELISA for Cytokines IL-4, 1L-10, IFN-γ and IL-2 in Lavage Fluid

Commercially available ELISA kits were used to assess expression of cytokines IL-4, 1L-10, IFN-γ and IL-2 in both NALF and BALF, according to the instructions of the manufactures. The standard curve range for mouse IL-4, 1L-10, IFN-γ and IL-2 were 4–500, 30–4000, 15–2000, and 2–200 pg/ml, respectively.

### Flow Cytometry Analysis for Regulatory T cells (Tregs)

In order to determine whether the immunomodulatory effects depend on Tregs (CD4^+^CD25^+^FoxP3^+^), percentages of Tregs in paratracheal lymph nodes (PTLN) were determined by one step mouse Treg flow staining kit. PTLN cells were collected at the moment that mice were sacrificed after 24 h of the final challenge and then washed in PBS with 1% FBS. Each tube contained 100 µl of approximately 10^6^ prepared cells. Single cell suspensions were stained for surface molecules anti-mouse FITC-conjugated CD4 and PE-conjugated CD25 as usual, and then stained for intracellular PE-Cy5-conjugated Foxp3 or isotype control after freshly fixation and permeabilization. After staining, the cells were washed and resuspended in an appropriate volume of flow cytometry staining buffer, and subsequently analyzed by flow cytometry (BeckmanCoulter, USA) under instructions of the given protocol.

### Statistical Analysis

All statistical analyses were performed with SPSS v.13.0 (SPSS, USA), and diagrams were done with GraphPad Prism v.5.0 (GraphPad Software, USA). One-way analysis of variance (ANOVO) followed by Student-Newman-Keuls test was used for multiple comparisons of data with Gaussion distribution. The Mann-Whitney U test was applied for data with abnormal distribution. *P* value less than 0.05 was considered statistical significance.

## Results

### *E. coli* Administration Suppresses the Frequency of Allergic Symptoms

**Figure 2:**
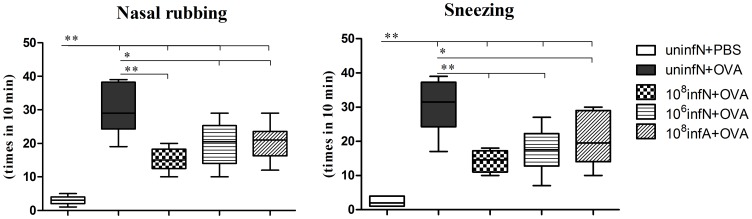
The changes of allergic symptoms. The control group showed no or less allergic symptoms, whereas AAD model group had remarkable frequency of nasal rubbing and sneezing. Nevertheless, the frequency was noticeably decreased by *E. coli* infection. Data is shown by box and whisker plots, with whisker ends indicating minimal and maximal values and horizontal bars representing medians, n = 10. ^*^ *p*<0.05, ^**^ *p*<0.01 as conducted.

In order to determine whether *E. coli* infection could affect the changes of allergic symptoms, we first measured the frequency of allergic symptoms per mouse in our work. The occurrences of nasal rubbing and sneezing per mouse in AAD model group were increased significantly, compared with that in the control (uninfN+PBS) group (*p*<0.01) (Fig. 2). Interestingly, we also found that oral *E. coli* treatment before the phase of AAD exhibited a significant inhibitory effect in down-regulating numbers of allergic symptoms (*p*<0.01). There were no noteworthy differences of allergic symptoms among different approaches to oral *E. coli* administration.

### *E. coli* Administration Decreases OVA-induced Inflammation Cells in Both NALF and BALF

**Figure 3:**
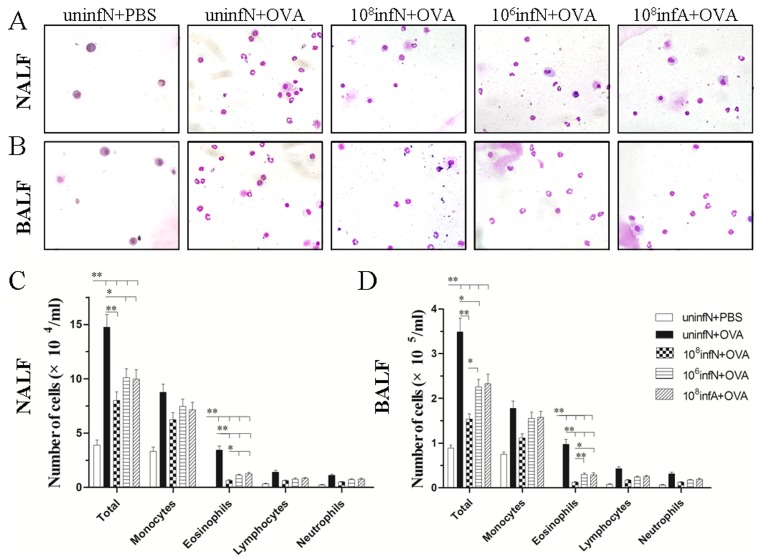
Absolute numbers of inflammation cells in 1.2 ml of NALF and BALF at 24 h after final challenge. Original magnification was ×400 (A, B). Total inflammation cells and eosinophils in NALF (C) and BALF (D) were significantly inhibited by *E. coli* infection in AAD mice model. Each bar represents the mean cell number ± standard error of the mean (SEM), n = 10. ^*^ *p*<0.05, ^**^ *p*<0.01 as conducted.

To better study the efficacy of oral *E. coli* administration before AAD phase, we next counted the inflammation cells obtained from NALF and BALF at the time of 24 h after the final challenge (Fig. 3A–B). More total inflammation cells as well as eosinophils in NALF and BALF were detectable in AAD model group than the control group (all *p*<0.01), along with increased numbers of other related cell types (monocytes, lymphocytes and neutrophils) (Fig. 3C–D). However, treatment with oral *E. coli* before AAD phase reduced numbers of total and eosinophil cells both in NALF and BALF (*p*<0.05 or *p*<0.01). Interestingly, the decrease of inflammation cells was most robust in mice neonatally infected with 10^8 ^CFU *E. coli*, compared to mice infected with 10^6 ^CFU or adultly infected (*p*<0.05 or *p*<0.01). These data suggested that oral *E. coli* administration had a potent suppressive effect on allergic symptoms, especially treated with a reasonable dose during the neonatal period.

### *E. coli* Administration Ameliorates OVA-induced Upper and Lower Allergic Airway Inflammation

Furthermore, we qualitatively and quantitatively examined the alteration of allergic airway inflammation by histological analysis of eosinophil inflammation and goblet cell metaplasia in the nasal mucosa and lung.

**Figure 4:**
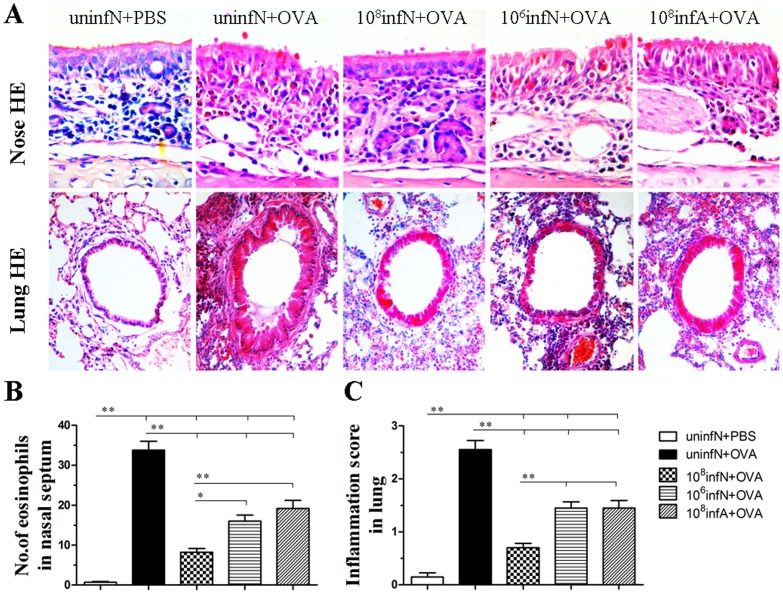
Eosinophil inflammation assessed on hematoxylin and eosin (HE) stained tissue sections of the nasal mucosa and lung. Original magnification was ×400 for nose and ×200 for lung (A). Numbers of eosinophils in the nasal mucosa (B) and inflammation scores of the lung (C) were counted to verify the inflammation changes among groups. Eosinophil infiltration was significantly higher in AAD model group than in the control group. Interestingly, *E. coli* infection before AAD phase drastically suppressed the eosinophil inflammation. In addition, numbers of eosinophils in the (10^8^infN+OVA) group were lower than the (10^6^infN+OVA) and (10^8^infA+OVA) group. Data is expressed as mean ±SEM, n = 10.^ *^ *p*<0.05, ^**^ *p*<0.01 as conducted.

**Figure 5:**
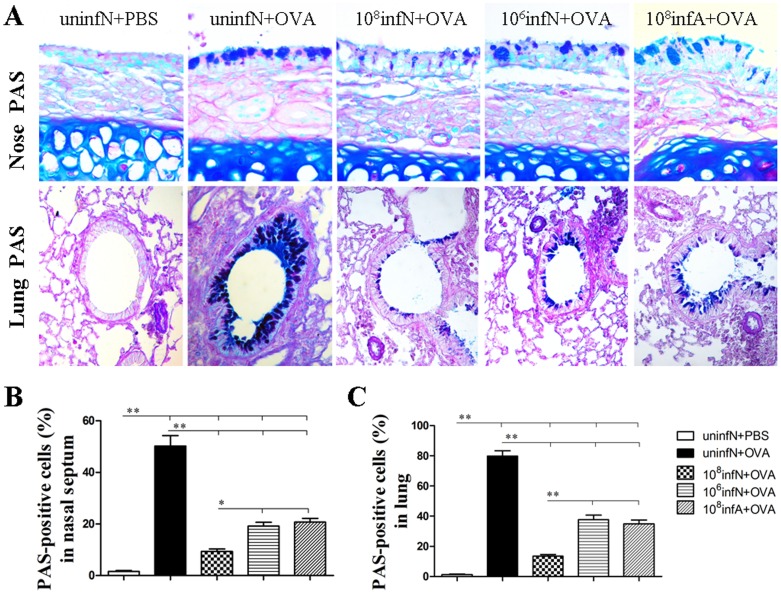
Goblet cell metaplasia assessed on alcian blue-periodic acid Stiff (AB-PAS) stained tissue sections of the nasal mucosa and lung. Original magnification was ×400 for nose and ×200 for lung (A). Goblet cells were counted as the blue cells stained positive by AB-PAS. Percentages of goblet cell metaplasia were calculated from the total numbers of cells counted around the nasal mucosa (B) and the lung (C). Goblet cell metaplasia was relatively minor in mice infected with *E. coli*. Data is expressed as mean percent ± SEM, n = 10.^ *^ *p*<0.05, ^**^ *p*<0.01 as conducted.

As shown in Fig. 4 and Fig. 5, there were no changes in the control group. However, there was a significant suppression of eosinophil infiltration (all *p*<0.01) (Fig. 4) and goblet cell metaplasia (all *p*<0.01) (Fig. 5) in the nasal mucosa and lung by oral *E. coli* administration. This indicated that oral *E. coli* administration before AAD phase had the ability to suppress OVA-induced allergic inflammation in both the upper and lower airways. Additionally in our study, in a comparison with (10^6^infN+OVA) group, (10^8^infN+OVA) group was found to present more ability in lowering eosinophil infiltration and goblet cell metaplasia in the nasal mucosa (both *p*<0.05) (Fig. 4B and Fig. 5B), as well as in the lung (both *p*<0.01) (Fig. 4C and Fig. 5C), which illustrated that AAD protection conferred by oral *E. coli* infection was probably dose-dependent. More importantly in our study, compared to the (10^8^infA+OVA) group, the (10^8^infN+OVA) group was detected to significantly reduce more allergic airway inflammation in the nasal mucosa (*p*<0.01 for eosinophil infiltration and *p*<0.05 for goblet cell metaplasia) (Fig. 4B and Fig. 5B), along with similar inhibitory effects in the lung (*p*<0.01 for both eosinophil infiltration and goblet cell metaplasia) (Fig. 4C and Fig. 5C), which inferred that AAD protection mediated by oral *E. coli* infection was also potentially age-dependent. Taken together, our study certified that the oral *E. coli* mediated-inhibited effects on the immune system might have a close internal sensitivity on the dose as well as the age.

### *E. coli* Administration Reduces Levels of Serum OVA-specific IgE

**Figure 6:**
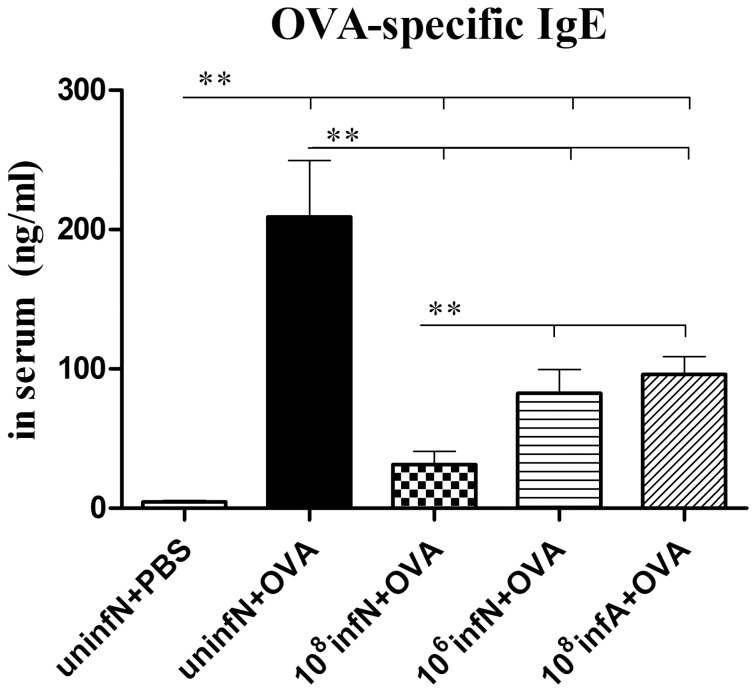
The changes of serum OVA-specific IgE levels. OVA-specific IgE levels were apparently higher in AAD model group than that in the control group. However, the levels in *E. coli* infected mice were significantly inhibited, especially in the (10^8^infN+OVA) group. Bars indicate the mean secretion ng/ml ± SEM, n = 8∼10.^ *^ *p*<0.05, ^**^ *p*<0.01 as conducted.

Although serum IgE level alone did not entirely reflect the allergic state and the clinical symptoms, it was apparent that a raised level of IgE represented the Th2 immune response and helped aid the diagnosis of allergic disease [5]. Hence we analyzed OVA-specific IgE levels in the serum prepared from blood 24 h after the final challenge. Study showed that levels of serum OVA-specific IgE were significantly higher in AAD model group than that in the control group (*p*<0.01) (Fig. 6). However, oral *E. coli* administration markedly suppressed the circulating IgE levels. Furthermore, our study also revealed that the neonatal infection with 10^8 ^CFU *E. coli* significantly decreased more OVA-specific IgE levels, in contrast to oral *E. coli* administration with 10^6 ^CFU dose or during adult period (both *p*<0.01), which was a coincidence with the results of histological analysis.

### *E. coli* Administration Alters Cytokine Production in both NALF and BALF

**Figure 7:**
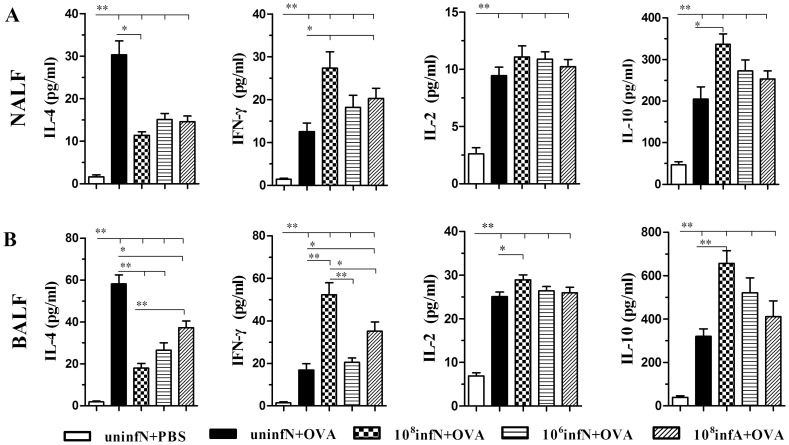
The changes of cytokines IL-4, IL-10, IFN-γ and IL-2 in NALF (A) and BALF (B). As shown above, administration of *E. coli* exhibited significant inhibition of levels of Th2 cytokines IL-4. Interestingly, the effect was accompanied by high levels of Th1 cytokines IFN-γ and IL-2, as well as the increased production of IL-10 secreted abundantly by Tregs. Additionally, the effects were more significant in neonatal mice infected with 10^8^CFU *E. coli*. Data is represented as the mean secretion pg/ml ± SEM, n = 8∼10.^ *^ *p*<0.05, ^**^ *p*<0.01 as conducted.

To further investigate the skewing of Th2-specific immune responses conferred by oral *E. coli*, we measured cytokines IL-4, IFN-γ, IL-2 and IL-10 levels in both NALF and BALF (Fig. 7). NALF and BALF from AAD model group both contained obviously detectable levels of Th2 cytokines IL-4 compared with the control group (*p*<0.01), while *E. coli* treatment groups, especially mice neonatally infected with 10^8 ^CFU, contained relative low levels compared with AAD model group (*p*<0.05 or *p*<0.01). In contrast with Th2 cytokines IL-4, levels of Th1 cytokines IFN-γ and IL-2, particularly IFN-γ, were significantly higher in mice neonatally infected 10^8 ^CFU *E. coli* than that in AAD model group (*p*<0.05 for IFN-γ in NALF, *p*<0.01 for IFN-γ in BALF, and *p*<0.05 for IL-2 in BALF), along with the increase in groups of mice infected with 10^6 ^CFU or during adults though not so significant (*p*<0.05 for IFN-γ in both NALF and BALF of (10^8^infA +OVA) group). Immune response of *E. coli* to AAD was likewise observed on levels of IL-10, which is abundantly secreted by Tregs to mediate immune suppression, and also a pleiotropic cytokine released by Th1 and Th2 cells as well [31]. Considerably increased production of IL-10 was observed in mice neonatally infected with 10^8 ^CFU *E. coli*, compared to AAD model group (*p*<0.05 for NALF, and *p*<0.01 for BALF).

### *E. coli* Administration Up-regulates Production of IL-10-secreting Tregs in PTLN

**Figure 8:**
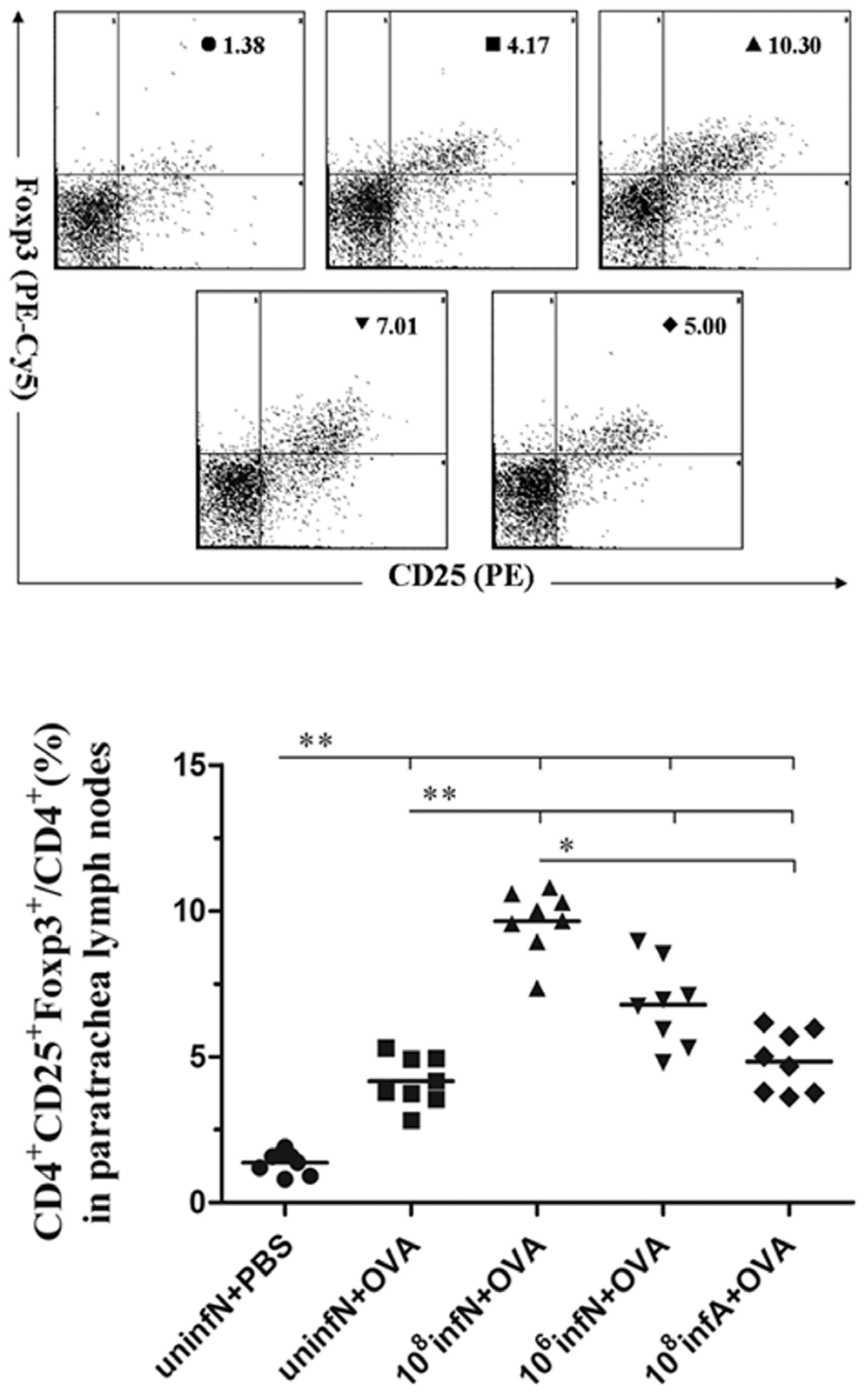
Tregs accumulated in PTLN of *E. coli* infected mice, especially in the (10^8^infN+OVA) group. Representative scatter plots denoted the fraction of CD4^+^ cells that were CD4^+^ CD25^+^ FoxP3^+^ Tregs (A). Ratios of Tregs were calculated per mouse (B). Percentages of Tregs in AAD model group were increased, compared to the control group. Interestingly, mice infected with *E. coli* present a more significant up-regulation in numbers of Tregs. Additionally, numbers of Tregs in the (10^6^infN+OVA) and (10^8^infA+OVA) group were lower than that in the (10^8^infN+OVA) group. Data is expressed as mean ± SEM, n = 8.^ *^ *p*<0.05, ^**^ *p*<0.01 as conducted.

To better investigate whether *E. coli* treatment induced production of Tregs and to evaluate the role of Tregs in the suppression of AAD, we assayed the accurate percentages of CD4^+^CD25^+^Foxp3^+^ Tregs in PTLN at time that mice were sacrificed after 24 h of the final challenge (Fig. 8). Percentages of Tregs in CD4^+^ cells were comparable between AAD model group and the control group (*p*<0.01). Interestingly, in comparison with AAD model group, mice infected with *E. coli* before AAD phase possessed more significant potential for up-regulation of numbers of Tregs (all *p*<0.01), which had a potent suppressive capacity through secretion of IL-10. Moreover, numbers of Tregs in mice neonatally infected with 10^8 ^CFU *E. coli* were higher than that in mice infected with 10^6 ^CFU or adultly infected (*p*<0.05). Above data indicated that certain dose- and age-sensitivity of *E. coli* exposure was critical for establishing adequate Tregs to regulate our immune system in terms of preventing AAD.

## Discussion

An increasing number of evidence has proclaimed that the upper and the lower airways share most common pathologies and mechanisms [3], [4]. In our study, we succeeded in developing a new mouse model of allergic airway inflammation in both the nasal mucosa and the lung induced by OVA according to previous reports with minor modification [25], [26], which exhibited frequent nasal rubbing and sneezing, abundant eosinophil infiltration and goblet cell metaplasia into the airway mucosa, excessive specific IgE levels, and Th2 skewing of the immune response.

Allergic rhinitis and asthma have been increasing worldwide leading to global financial and substantial medical burdens [1]–[2], as yet, there has still been no effective pattern of therapy so far. Nevertheless, reams of evidence currently being investigated have demonstrated that certain environmental factors could attenuate the allergic responses in allergic rhinitis and/or asthma [32]. Numbers of microorganisms that colonize on mammalian body surfaces have a highly close relationship with the immune system. The resident microbiota, such as certain bacteria, helminthes and so forth [33]–[36], has profoundly shaped mammalian immunity, the immunomodulatory potential of which has made them promising candidates for allergic disease therapy. More recently, there is still a gap for a body evidence to elucidate the immunomodulatory function of our main and most common gut microflora-*E. coli*, although certain epidemiological ones have been operated [19], [20], [37], [38]. Hence in our study, we utilized administration of nonpathogenic *E. coli* ATCC 25922 before the OVA sensitization and challenged phase, and assessed whether *E. coli* ATCC 25922 was able to either suppress the induction of allergic airway inflammation or regulate the immune responses.

Our study, for the first time, showed that *E. coli* infection suppressed the hallmark features of the allergic responses. *E. coli* infection before AAD phase, significantly attenuated allergic symptoms of nasal rubbing and sneezing, decreased the pathology of eosinophil infiltration and goblet cell metaplasia in the nasal mucosa and lung, inhibited serum OVA-specific IgE levels, and suppressed levels of Th2 cytokines in NALF and BALF. These data confirmed the hygiene hypothesis that the lack of bacterial infections, including the gut microflora, would favour the development of allergic disease. More importantly for the rigidity of the experiment, the above findings were profoundly confirmed by the three patterns of intragastric administration of *E. coli*, thus illustrating their stable capability to regulate the immune responses during allergic airway inflammation. To our knowledge, in the low intestine, *E. coli* and other variety of gut microflora colonized to an extraordinary density and evolved to degrade kinds of plant polysaccharides and other related dietary substances [39]. Not only has it enhanced the host digestive efficiency and ensured a steady nutrient supply for microbes, but also probably driven the evolution of the immune system, thus protecting the host from the pathogens and regulating the induction of immunological tolerance [13], [19]. For this reason, it was plausible that *E. coli* infection possessed a formidable potential immunomodulatory function to allergic disease. In the analysis of fecal stool flora in our study, we detected that *E. coli* neonatally infected mice had a statistically noteworthy increase in the total number of normal intestinal flora especially in the quantities of enterobacteria, compared to that of the uninfected neonatal mice, by identifying patterns of Gram-stained fecal flora and using molecular detection methods. These changes might be associated with infectious disease in a healthy body, but potentially beneficial in the context of an allergic surrounding. Furthermore, we didn’t find the appearance of any enteric pathogens in the fecal microbiota of *E. coli* infected mice, such as Staphylococcus aureus, *Pseudomonas aeruginosa, Klebsiella pneumoniae, Proteus mirabilis* and so forth (data not shown). As far as we know, it was the first time for the present study to investigate the potential role of *E. coli* in the modulation of allergic responses in a mouse model.

So what is the underlying mechanism? Data in this work declared that *E. coli* infection inhibited eosinophil inflammation probably partly via shifting to a Th1 from a Th2 immune response to the OVA allergen. In this experiment, we discriminated Th1/Th2 subsets based on the expression of Th2 cytokines IL-4, Th2-like immune responses of OVA-specific IgE, as well as Th1 cytokines IFN-γ and IL-2, which were assayed accurately by ELISA. Our work showed that levels of IL-4 and OVA-specific IgE in AAD model group were significantly increased, thus clarifying that allergic inflammation was mediated by the Th2 immune response. However, *E. coli* infection before AAD phase suppressed Th2 cytokines IL-4 and serum IgE production, but in contrast enhanced Th1 cytokines IFN-γ and IL-2 production, Moreover, this effects were more significantly in the (10^8^infN+OVA) group than the (10^6^infN+OVA) and (10^8^infA+OVA) group. Similar to our study, mounting previous reports [5]–[7] have confirmed that AAD was an aberrant Th2 cytokine mediated immune response, and Th2 cytokines induced eosinophil inflammation. In terms of immune responses, Th1 and Th2 cytokines are mutually antagonistic [40]. Selective up-regulation of a Th1 and down-regulation of a Th2 immune response might be crucial for the prevention of allergic airway inflammation [41], although the present study could not be fully explained the exact mechanism of the skewing from a Th2 to a Th1 response, which was also the limitations of our study.

Another potential mechanism was also observed in our study. We found that percentages of CD4^+^CD25^+^Foxp3^+^ Tregs in CD4^+^ PTLN cells were significantly elevated by *E. coli* infection, along with the enhanced IL-10 secretion. Further more, conspicuous differences were also observed in numbers of IL-10-secreting Tregs among the three *E. coli* infection groups. These data were consistent with previous studies [35], [42], [43] in which Tregs were conferred to expand in the draining lymph nodes before moving to the inflammatory site and to play an immunosuppressive role in the protection against allergic disorders. Notably, IL-10 is an immunosuppressive cytokine that may be released mainly by Tregs to mediate suppression, and also a pleiotropic cytokine released by Th1 and Th2 cells as well in AAD [31], [35], [41]. Further investigations are underway to further characterize the role of IL-10-secreting Tregs and to elucidate other possible mechanisms of *E. coli*-mediated suppression of AAD.

Interestingly, detectable differences were observed on the efficacy of the three means of *E. coli* administration, including the frequency of nasal rubbing and sneezing, numbers of inflammation cells, serum levels of OVA-specific IgE, production of Th1 and Th2 cytokines, as well as numbers of accumulated Tregs. *E. coli* infection in the (10^8^infN+OVA) group, which was administrated in a neonatal age and an optimal dose, showed more strength of anti-inflammation than the (10^6^infN+OVA) and (10^8^infA+OVA) group. These results indicate that *E. coli*-mediated protection was age- and dose-dependent and was critical for establishing mature immune systems to later environmental exposures, which agreed with a previous study that an optical dose was more effective than an improper dose for microbial infection [44], and also agreed with recent studies in which microbial exposure during neonatal or early life had persistent and durable immunomodulatory effects, but not adults [35], [45].

To our knowledge, hygiene hypothesis has demonstrated that, in recent decades, the increasing prevalence of allergic rhinitis and asthma is closely linked to the overly hygienic lifestyle and excessive use of antibiotics [8]–[11], [46]–[48], which might attribute to growing lack of commensal microbes stimuli. For this reason, our ancestral indigenous gut microflora or their components could be used as potential candidates for allergic diseases. Similar to our study, growing evidence has thus come to the technology known as “the good the gut bugs do” [16]. We do not recommend the consumption of microbial food including *E. coli*, but promote our earlier trend in touch with nature and in contact with the rural lifestyle, as well as prompt a reasonable clinical application of antibiotics, rather than abuse. These complex microbial-host interactions operate in a delicate temporal and spatial manner and have an essential role in the induction of homeostatic mechanisms, which requires interactions with the gut microflora [8]–[17].

In conclusion, we demonstrated, for the first time, that *E. coli* infection prior to allergen sensitization had shown promise in inducing immune tolerance, probably via a shift from a Th2 to a Th1 immune response and/or induction of local Tregs. Our findings may open up possibilities that interactions with *E. coli* microflora may be used as an alternative strategy for prevention of AAD.

## References

[1] Bousquet J, Khaltaev N, Cruz AA, Denburg J, Fokkens WJ, et al (2008) Allergic Rhinitis and its Impact on Asthma (ARIA) 2008 update (in collaboration with the World Health Organization, GA(2)LEN and AllerGen). Allergy 63 Suppl 86 8–160. 18331513 10.1111/j.1398-9995.2007.01620.x

[2] Brozek JL, Bousquet J, Baena-Cagnani CE, Bonini S, Canonica GW, et al (2010) Allergic Rhinitis and its Impact on Asthma (ARIA) guidelines: 2010 revision. J Allergy Clin Immunol 126(3): 466–476. 20816182 10.1016/j.jaci.2010.06.047

[3] Compalati E, Ridolo E, Passalacqua G, Braido F, Villa E, et al (2010) The link between allergic rhinitis and asthma: the united airways disease. Expert Rev Clin Immunol 6(3): 413–423. 20441427 10.1586/eci.10.15

[4] Galli SJ, Tsai M, Piliponsky AM (2008) The development of allergic inflammation. Nature 454(7203): 445–454. 18650915 10.1038/nature07204 PMC3573758

[5] Wang D-Y, Clement P (2000) Pathogenic mechanisms underlying the clinical symptoms of allergic rhinitis. Am J Rhinol 14(5): 325–33. 11068658 10.2500/105065800781329483

[6] Lukacs NW (2001) Role of chemokines in the pathogenesis of asthma. Nat Rev Immunol 1(2): 108–116. 11905818 10.1038/35100503

[7] Nauta AJ, Engels F, Knippels LM, Garssen J, Nijkamp FP, et al (2008) Mechanisms of allergy and asthma. Eur J Pharmacol 585(2–3): 354–360. 18410921 10.1016/j.ejphar.2008.02.094

[8] Strachan DP (1989) Hay fever, hygiene, and household size. BMJ 299(6710): 1259–1260. 2513902 10.1136/bmj.299.6710.1259 PMC1838109

[9] Eder W, Ege MJ, von Mutius E (2006) The asthma epidemic. N Engl J Med 355(21): 2226–2235. 17124020 10.1056/NEJMra054308

[10] Schaub B, Lauener R, von Mutius E (2006) The many faces of the hygiene hypothesis. J Allergy Clin Immunol 117(5): 969–977. 16675321 10.1016/j.jaci.2006.03.003

[11] Okada H, Kuhn C, Feillet H, Bach JF (2010) The 'hygiene hypothesis' for autoimmune and allergic diseases: an update. Clin Exp Immunol 160(1): 1–9. 10.1111/j.1365-2249.2010.04139.x PMC2841828 20415844

[12] Mayer M, Normand AC, Genuneit J, Cookson WO, Braun-Fahrlander C, et al (2011) Exposure to environmental microorganisms and childhood asthma. N Engl J Med 364(8): 701–709. 21345099 10.1056/NEJMoa1007302

[13] Hooper LV, Littman DR, Macpherson AJ (2012) Interactions between the microbiota and the immune system. Science 336(6086): 1268–1273. 22674334 10.1126/science.1223490 PMC4420145

[14] Hill DA, Siracusa MC, Abt MC, Kim BS, Kobuley D, et al (2012) Commensal bacteria-derived signals regulate basophil hematopoiesis and allergic inflammation. Nat Med 18(4): 538–546. 22447074 10.1038/nm.2657 PMC3321082

[15] Blaser MJ, Falkow S (2009) What are the consequences of the disappearing human microbiota? Nat Rev Microbiol 7(12): 887–894. 19898491 10.1038/nrmicro2245 PMC9354563

[16] Leavy O (2012) Mucosal immunology: the good the gut bugs do. Nat Rev Immunol 12(5): 319. 22498779 10.1038/nri3213

[17] Renz H, Brandtzaeg P, Hornef M (2011) The impact of perinatal immune development on mucosal homeostasis and chronic inflammation. Nat Rev Immunol 12(1): 9–23. 22158411 10.1038/nri3112

[18] Mackie RI, Sghir A, Gaskins HR (1999) Developmental microbial ecology of the neonatal gastrointestinal tract. Am J Clin Nutr 69(5): 1035S–1045S. 10232646 10.1093/ajcn/69.5.1035s

[19] Wold AE (1998) The hygiene hypothesis revised: is the rising frequency of allergy due to changes in the intestinal flora? Allergy 53(46 Suppl): 20–25. 10.1111/j.1398-9995.1998.tb04953.x 9825991

[20] Boudeau J, Glasser AL, Julien S, Colombel JF, Darfeuille-Michaud A (2003) Inhibitory effect of probiotic Escherichia coli strain Nissle 1917 on adhesion to and invasion of intestinal epithelial cells by adherent-invasive *E. coli* strains isolated from patients with Crohn's disease. Aliment Pharmacol Ther 18(1): 45–56. 10.1046/j.1365-2036.2003.01638.x 12848625

[21] Sharma V, Suvarna K, Meganathan R, Hudspeth ME (1992) Menaquinone (vitamin K2) biosynthesis: nucleotide sequence and expression of the menB gene from *Escherichia coli* . J Bacteriol 174(15): 5057–5062. 1629162 10.1128/jb.174.15.5057-5062.1992 PMC206321

[22] Lobry JR, Carret G, Flandrois JP (1992) Maintenance requirements of *Escherichia coli* ATCC 25922 in the presence of sub-inhibitory concentrations of various antibiotics. J Antimicrob Chemother 29(2): 121–127. 1506326 10.1093/jac/29.2.121

[23] Uri JV (1994) Is *Escherichia coli* ATCC 25922 a colicin producing strain? (a note). Acta Microbiol Immunol Hung 41(2): 215–219. 7804725

[24] Sauer A, Moraru CI (2009) Inactivation of *Escherichia coli* ATCC 25922 and *Escherichia coli* O157:H7 in apple juice and apple cider, using pulsed light treatment. J Food Prot 72(5): 937–944. 19517718 10.4315/0362-028x-72.5.937

[25] Sun YQ, Deng MX, He J, Zeng QX, Wen W, et al (2012) Human pluripotent stem cell-derived mesenchymal stem cells prevent allergic airway inflammation in mice. Stem Cells 30(12): 2692–2699. 22987325 10.1002/stem.1241 PMC3549478

[26] Hellings PW, Hessel EM, Van Den Oord JJ, Kasran A, Van Hecke P, et al (2001) Eosinophilic rhinitis accompanies the development of lower airway inflammation and hyper-reactivity in sensitized mice exposed to aerosolized allergen. Clin Exp Allergy 31(5): 782–790. 11422139 10.1046/j.1365-2222.2001.01081.x

[27] Yokota E, Kuyama S, Ogawa M, Kamei C (2008) Substance P is involved in the effect of histamine H3 receptor agonist, Sch 50971 on nasal allergic symptoms in mice. Int Immunopharmacol 8(8): 1083–1088. 18550011 10.1016/j.intimp.2008.03.018

[28] Zhu D, Kepley CL, Zhang K, Terada T, Yamada T, et al (2005) A chimeric human-cat fusion protein blocks cat-induced allergy. Nat Med 11(4): 446–449. 15793580 10.1038/nm1219

[29] Kang JH, Kim BS, Uhm TG, Lee SH, Lee GR, et al (2009) Gamma-secretase inhibitor reduces allergic pulmonary inflammation by modulating Th1 and Th2 responses. Am J Respir Crit Care Med 179(10): 875–882. 19234107 10.1164/rccm.200806-893OC

[30] Henderson WR Jr, Banerjee ER, Chi EY (2005) Differential effects of (S)- and (R)-enantiomers of albuterol in a mouse asthma model. J Allergy Clin Immunol 116(2): 332–340. 16083788 10.1016/j.jaci.2005.04.013

[31] Akdis M, Burgler S, Crameri R, Eiwegger T, Fujita H, et al (2011) Interleukins, from 1 to 37, and interferon-γ: receptors, functions, and roles in diseases. J Allergy Clin Immunol 127(3): 701–721. 21377040 10.1016/j.jaci.2010.11.050

[32] Robinson CJ, Bohannan BJ, Young VB (2010) From structure to function: the ecology of host-associated microbial communities. Microbiol Mol Biol Rev 74(3): 453–76. 20805407 10.1128/MMBR.00014-10 PMC2937523

[33] Shirakawa T, Enomoto T, Shimazu S, Hopkin JM (1997) The inverse association between tuberculin responses and atopic disorder. Science 275(5296): 77–79. 8974396 10.1126/science.275.5296.77

[34] Yazdanbakhsh M, Kremsner PG, van Ree R (2002) Allergy, parasites, and the hygiene hypothesis. Science 296(5567): 490–494. 11964470 10.1126/science.296.5567.490

[35] Arnold IC, Dehzad N, Reuter S, Martin H, Becher B, et al (2011) Helicobacter pylori infection prevents allergic asthma in mouse models through the induction of regulatory T cells. J Clin Invest 121(8): 3088–3093. 21737881 10.1172/JCI45041 PMC3148731

[36] Qiu H, Kuolee R, Harris G, Zhou H, Miller H, et al (2011) Acinetobacter baumannii infection inhibits airway eosinophilia and lung pathology in a mouse model of allergic asthma. PLoS One 6(7): e22004. 21789200 10.1371/journal.pone.0022004 PMC3138758

[37] Savilahti EM, Kukkonen AK, Haahtela T, Tuure T, Kuitunen M, et al. Intestinal defensin secretion in infancy is associated with the emergence of sensitization and atopic dermatitis. Clin Exp Allergy 42(3): 405–411. 22093109 10.1111/j.1365-2222.2011.03904.x

[38] Rose MA, Weigand B, Schubert R, Schulze J, Zielen S (2011) Safety, tolerability, and impact on allergic inflammation of autologous *E. coli* autovaccine in the treatment of house dust mite asthma-a prospective open clinical trial. BMC Complement Altern Med 11: 45. 21639872 10.1186/1472-6882-11-45 PMC3141598

[39] Ley RE, Lozupone CA, Hamady M, Knight R, Gordon JI (2008) Worlds within worlds: evolution of the vertebrate gut microbiota. Nat Rev Microbiol 6(10): 776–788. 18794915 10.1038/nrmicro1978 PMC2664199

[40] Skapenko A, Schulze-Koops H (2007) Analysis of Th1/Th2 T-cell subsets. Methods Mol Med 136: 87–96. 17983142 10.1007/978-1-59745-402-5_7

[41] Cho KS, Park HK, Park HY, Jung JS, Jeon SG, et al (2009) IFATS collection: Immunomodulatory effects of adipose tissue-derived stem cells in an allergic rhinitis mouse model. Stem Cells 27(1): 259–265. 18832595 10.1634/stemcells.2008-0283

[42] Robinson DS (2009) Regulatory T cells and asthma. Clin Exp Allergy 126(6): 1314–1323. 10.1111/j.1365-2222.2009.03301.x 19538496

[43] Thorburn AN, O'Sullivan BJ, Thomas R, Kumar RK, Foster PS, et al (2010) Pneumococcal conjugate vaccine-induced regulatory T cells suppress the development of allergic airways disease. Thorax 65(12): 1053–1060. 20965927 10.1136/thx.2009.131508

[44] Smit JJ, Van Loveren H, Hoekstra MO, Schijf MA, Folkerts G, et al (2003) Mycobacterium vaccae administration during allergen sensitization or challenge suppresses asthmatic features. Clin Exp Allergy 33(8): 1083–1089. 12911782 10.1046/j.1365-2222.2003.01727.x

[45] Olszak T, An D, Zeissig S, Vera MP, Richter J, et al (2012) Microbial exposure during early life has persistent effects on natural killer T cell function. Science 336(6080): 489–493. 22442383 10.1126/science.1219328 PMC3437652

[46] Jenerowicz D, Silny W, Danczak-Pazdrowska A, Polanska A, Osmola-Mankowska A, et al (2012) Environmental factors and allergic diseases. Ann Agric Environ Med 19(3): 475–481. 23020042

[47] Russell SL, Gold MJ, Hartmann M, Willing BP, Thorson L, et al (2012) Early life antibiotic-driven changes in microbiota enhance susceptibility to allergic asthma. EMBO Rep 13(5): 440–447. 22422004 10.1038/embor.2012.32 PMC3343350

[48] Renz H, Brandtzaeg P, Hornef M (2011) The impact of perinatal immune development on mucosal homeostasis and chronic inflammation. Nat Rev Immunol 12(1): 9–23. 22158411 10.1038/nri3112

